# Infection with Soil-Transmitted Helminths Is Associated with Increased Insulin Sensitivity

**DOI:** 10.1371/journal.pone.0127746

**Published:** 2015-06-10

**Authors:** Aprilianto E. Wiria, Firdaus Hamid, Linda J. Wammes, Margaretta A. Prasetyani, Olaf M. Dekkers, Linda May, Maria M. M. Kaisar, Jaco J. Verweij, Bruno Guigas, Felix Partono, Erliyani Sartono, Taniawati Supali, Maria Yazdanbakhsh, Johannes W. A. Smit

**Affiliations:** 1 Department of Parasitology, Faculty of Medicine, Universitas Indonesia, 10430, Jakarta, Indonesia; 2 Department of Parasitology, Leiden University Medical Center, 2333ZA, Leiden, The Netherlands; 3 Department of Microbiology, Faculty of Medicine, Hasanuddin University, 90245, Makassar, Indonesia; 4 Department of Clinical Epidemiology, Leiden University Medical Center, 2333ZA, Leiden, The Netherlands; 5 Department of Endocrinology & General Internal Medicine, Leiden University Medical Center, 2333ZA, Leiden, The Netherlands; 6 Laboratory for Medical Microbiology and Immunology, St. Elisabeth Hospital, 5022GC, Tilburg, The Netherlands; 7 Department of Molecular Cell Biology, Leiden University Medical Center, 2333ZA, Leiden, The Netherlands; 8 Department of General Internal Medicine, Radboud University Medical Center, 6525GA, Nijmegen, The Netherlands; University of Leipzig, GERMANY

## Abstract

**Objective:**

Given that helminth infections have been shown to improve insulin sensitivity in animal studies, which may be explained by beneficial effects on energy balance or by a shift in the immune system to an anti-inflammatory profile, we investigated whether soil-transmitted helminth (STH)-infected subjects are more insulin sensitive than STH-uninfected subjects.

**Design:**

We performed a cross-sectional study on Flores island, Indonesia, an area with high prevalence of STH infections.

**Methods:**

From 646 adults, stool samples were screened for *Trichuris trichiura* by microscopy and for *Ascaris lumbricoides*, *Necator americanus*, *Ancylostoma duodenale*, *and Strongyloides stercoralis* by qPCR. No other helminth was found. We collected data on body mass index (BMI, kg/m^2^), waist-to-hip ratio (WHR), fasting blood glucose (FBG, mmol/L), insulin (pmol/L), high sensitive C-reactive protein (ng/ml) and Immunoglobulin E (IU/ml). The homeostatic model assessment for insulin resistance (HOMAIR) was calculated and regression models were used to assess the association between STH infection status and insulin resistance.

**Results:**

424 (66%) participants had at least one STH infection. STH infected participants had lower BMI (23.2 vs 22.5 kg/m^2^, p value = 0.03) and lower HOMAIR (0.97 vs 0.81, p value = 0.05). In an age-, sex- and BMI-adjusted model a significant association was seen between the number of infections and HOMAIR: for every additional infection with STH species, the HOMAIR decreased by 0.10 (p for linear trend 0.01). This effect was mainly accounted for by a decrease in insulin of 4.9 pmol/L for every infection (p for trend = 0.07).

**Conclusion:**

STH infections are associated with a modest improvement of insulin sensitivity, which is not accounted for by STH effects on BMI alone.

## Introduction

The prevalence of type 2 diabetes (T2DM) is rising in low-to-middle income countries (LMIC). The explanations for these trends are complex and multifactorial, but have traditionally been attributed to a shift in infrastructure, technology and food supply that promotes over-nutrition and sedentary lifestyles [1, 2]. There is now accumulating evidence that in addition to a disturbed energy balance, inflammation plays a role in T2DM [3]. Indeed, in T2DM, elevated levels of inflammation-related markers such as interleukin 6 (IL6), IL8, tumor necrosis factor (TNF), and C-reactive protein (CRP) have been reported [3]. The adoption of a Western lifestyle in LMIC is often paralleled by decreased burden of infectious diseases, including helminth infections. Studying these trends in the context of the epidemiological transition theory and the hygiene hypothesis may have important implications for clinical practice, global health policy, and future research within epidemiology [4].

In animal models, helminth infections can enhance glucose tolerance, probably by inducing eosinophilia and preventing obesity [5]. Interestingly, helminth infections have been shown to induce T helper (Th) 2 cells [6–8] and anti-inflammatory immune responses [9–12]. It has been hypothesized that chronic helminth infections decrease systemic inflammation [13] and might be beneficial for the prevention of inflammatory diseases, such as allergy [11,14], inflammatory bowel disease [15], and T2DM [5,10,16]. Based on this information, it could be hypothesized that helminth infections may also have a beneficial influence on glucose metabolism both by preventing obesity and by anti-inflammatory immune responses [16]. In several epidemiological studies, an inverse association between the prevalence of T2DM or metabolic syndrome and helminth infections was found [17–19].

In the present study, we investigated the relationship between helminth infections and insulin resistance, as assessed by homeostatic model assessment for insulin resistance (HOMAIR) [20] in an area endemic for STH on Flores Island, Indonesia. In addition, we studied whether the potential association is explained by changes in body mass index (BMI).

## Subjects and Methods

### Study objectives

The primary objective of the study was to investigate the relationship between STH infections and HOMAIR in adults. Our hypothesis is that HOMAIR is lower in subjects with STH infections than in subjects without STH infections. This hypothesis is based on the association between helminth infections and decreased food intake, digestion or nutrient absorption, which will lead to lower BMI [21–23] as well as the proposed effects of helminth infections on systemic inflammation [13]. We studied to what extent the potential relationship between STH infections and HOMAIR is explained by differences BMI.

### Study population

The study area is Nangapanda on Flores Island in Indonesia, which is part of East Nusa Tenggara province. The area has a low socioeconomic status according to the recent Indonesian health survey (RISKESDAS 2013). Based on the same survey, prevalence of T2DM in this area was 3.3% in 2013. Previous reports from the area [24,25], have indicated that prevalence of metabolic syndrome according to ATPIII criteria is 11.8% (7.4% male, 14.4% female) [26,27]. Main sources of income are farming, fishing, woving and stone collection with a diet mainly consisting of freshly grown vegetables and fresh fish [28]. The area is highly endemic for STH, whereas no evidence for other helminth species has been found [28,29]. In Nangapanda area, a large investigational project is being conducted on the relationship between STH infections and the immune system (ImmunoSPIN study [28,29]). For the current study, a cross sectional sample was included from all inhabitants aged 18 years and above. Data were collected between May-August 2009.

### Study design

From 1841 inhabitants aged 18 years and above in Nangapanda who participated in the ImmunoSPIN project, 646 subjects from whom stool samples were available, were invited to participate in the present cross-sectional study for collection of data on anthropometrics and laboratory measurements. 584 subjects, from whom data on STH infections, BMI, waist-to-hip ratio (WHR) and laboratory measurements were available were included in the present analysis.

The study was approved by the ethical committee of the Faculty of Medicine, University of Indonesia (EC-FMUI), ref: 194/PT02.FK/Etik/2006 with addendum ref: 96/PT02.FK/Etik/2010 and registered as clinical trial ref: ISRCTN83830814 and was filed by the Leiden University Medical Center Committee of Medical Ethics (CME). Because of the high rate of illiteracy amongst elderly participants, either written or verbal informed consent (recorded as signed or with a given thumb-print) was obtained from each participant after explanation of the study and the voluntary nature of the participation.

### Clinical and laboratory assessments

Anthropometric measurements of body weight (SECA 761, SECA GMBH & Co. Kg., Hamburg, Germany), height (SECA 206, SECA GMBH & Co. Kg., Hamburg, Germany), waist and hip circumference (SECA 203, SECA GMBH & Co. Kg., Hamburg, Germany) were performed according to the NHLBI practical guidelines (NHLBI web: http://www.nhlbi.nih.gov) by a team of trained researchers. BMI was calculated as weight in kg divided by square of height in meter; WHR was calculated as waist circumference in cm divided by hip circumference in cm.

Participants were instructed to fast after 8 pm the day before blood collection. Fasting blood glucose (FBG) was analyzed using Breeze2 glucose meter (Bayer Health Care LLC, Basel, Switzerland). Insulin was measured using MSD 96-Well MULTI-ARRAY Human insulin assay (Meso Scale Discovery, Gaithersburg, USA). HOMAIR, a well-validated measure of IR, HOMAIR = fasting serum insulin x fasting glucose / 22.5, was calculated to estimate insulin resistance using HOMA2 calculator (https://www.dtu.ox.ac.uk/homacalculator/) [20]. High sensitive C-reactive protein (HsCRP) level was measured using MSD 96-Well MULTI-ARRAY CRP Assay (Meso Scale Discovery, Gaithersburg, USA).

Whole blood cytokine production after stimulation with *Escherichia coli* lipopolysaccharide (LPS) was performed as described previously [28]. Briefly, heparinized blood was diluted 4x and stimulated within 6 hours after drawing with medium alone or *E*. *coli* LPS, 1 ng/L Sigma-Aldrich, Zwijndrecht, The Netherlands) and incubated for 24 hours at 37^°^C and 5% CO_2_. The supernatants were frozen at -20^°^C and TNF and IL10 supernatants were assessed by means of immunobead-based multiplex assays on a Liquichip 200 Workstation (Qiagen, Venlo, The Netherlands) using Liquichip analyzer software (Qiagen, Venlo, The Netherlands). Samples with TNF levels higher than 250 pg/mL in medium stimulation (unstimulated blood) were excluded from further analyses (2 samples) as they are considered unreliable. Immunoglobulin E (IgE) level was measured by an ELISA [30].

### Assesment of soil-transmitted helminth infection

Stool samples were collected and preserved in 4% formaldehyde for microscopy examination or frozen (-20°C) unpreserved for PCR detection. The formol-ether acetate concentration method was performed on the formalin preserved stool samples followed by microscopy examination for eggs of STH [28]. For this paper only microcopy results on *Trichuris trichiura* infection were used as no PCR technique yet available. As described in detail before [28], DNA was isolated from approximately 100 mg unpreserved feces and a multiplex real-time PCR for the detection of *Ascaris lumbricoides*, *Necator americanus*, *Ancylostoma duodenale*, and *Strongyloides stercoralis* was performed. The real-time PCR output from this system consisted of a cycle-threshold (CT) value, representing the amplification cycle in which the level of fluorescent signal exceeds the background fluorescence, and reflecting the parasite-specific DNA load in the sample tested. Negative and positive control samples were included in each run of the amplification. We have indeed the result of stool cultured (Harada Mori) for detection of hookworm larvae. We have chosen to use PCR results whenever possible because this technique is proven to be very sensitive and specific [31]. We defined a positive case for *T*. *trichiura* by presence of eggs in stool samples and for *A*. *lumbricoides*, *N*. *americanus*, *A*. *duodenale* and *S*. *stercoralis* by parasite-specific DNA amplification. Participants were also stratified by number of STH species infections.

### Statistical analysis

Normally distributed continuous data were reported as mean and standard deviation. Normal distribution was assessed by verifying data distribution in a histogram graph relative to a normal distribution line. Non-normally distributed continuous data were expressed as median and interquartile range (insulin, HOMAIR, hsCRP, cytokines, IgE). Categorical data were expressed as proportions. Non-normally distributed data were log-transformed for analyses.

Differences in study parameters between subjects with and without any STH infection were analysed by linear regression. In addition, we also analysed the potential association between the number of helminth species per subject and HOMAIR. In all analyses, we adjusted for age and gender. To assess whether potential differences or associations are mediated through an effect of STH on BMI, we also adjusted for BMI in a separate analysis. Differences in immune parameters and HOMAIR between infected and uninfected participants were reported as mean differences with 95% confidence intervals (95% CI). P values <0.05 were considered to be statistically significant. In a supplementary analysis (S1 Table), we investigated the relationship between BMI categorized according to reference values for Asian subjects [32] and FBG, insulin and HOMAIR. Normality test (histogram and Shapiro–Wilk test) and statistical analyses were performed with SPSS 17.0.2 (SPSS Inc., Chicago, Illinois, The USA).

## Results

### Characteristics of study participants

A total of 424 participants who were infected with at least one species of STH were compared to 222 uninfected participants (Table 1). Gender and age distribution were comparable between the 2 groups. The most prevalent STH species among the study subjects were *N*. *americanus* (51.7%), *A*. *lumbricoides* (21.8%) and *T*. *trichiura* (19.7%). The proportion of participants infected with *A*. *duodenale* (3.7%) and with *S*. *stercoralis* (0.6%) was clearly lower. 261 participants were infected with one STH species only, 124 with two STH species and 39 with 3 or more STH species. Intestinal protozoa found in the participants were *Blatocystis hominis* 15 (2.3%), *Entamoeba hystolitica* 6 (0.9%), *Entamoeba coli* 37 (5.7%), *Giardia lamblia* 3 (0.5%). These proportions were considered too low to be used for further statistical analyses. BMI distribution and the relationship with FBG and HOMAIR is provided in a S1 Table.

**Table 1:** Characteristics of the study population.

	Whole study population	No infection with soil transmitted helminths	Infection with soil-transmitted helminths	P-value for difference infected vs non-infected
	(n = 646)	(n = 222)	(n = 424)	
Age (year) (mean, SD)	44.9 (13.9)	44.4 (13.2)	45.2 (14.2)	0.48
Female, N (%)	410 (63.5)	147 (66.2)	263 (62.0)	0.29
*Trichuris trichiura* N (%)	127 (19.7)		127 (30.0)	
*Ascaris lumbricoides* N (%)	141 (21.8)		141 (33.3)	
*Necator americanus* N (%)	334 (51.7)		334 (78.8)	
*Ancylostoma duodenale* N (%)	24 (3.7)		24 (5.7)	
*Strongyloides stercoralis* N (%)	4 (0.6)		4 (0.9)	
Single STH species infection N (%)	261 (40.4)		261 (61.6)	
Two STH species infecton N (%)	124 (19.2)		124 (29.2)	
Three or more STH species infection N (%)	39 (6.0)		39 (9.2)	
BMI (Kg/m^2^) (mean, SD)	22.7 (3.8)	23.2 (3.7)	22.5 (3.8)	0.03
WHR (mean, SD)	0.88 (0.07)	0.89 (0.07)	0.88 (0.06)	0.08
FBG (mmol/L) (mean, SD)	5.90 (1.6)	5.92 (1.5)	5.88 (1.6)	0.76
Insulin (pmol/L) (mean, SD)	46.5 (55.3)	49.5 (43.7)	45.0 (60.1)	0.40
HOMAIR (Mean, SD)	0.86 (0.86)	0.97 (0.84)	0.81 (0.86)	0.05
HsCRP (ng/ml) (Median, interquartile range)	469 (185–1354)	437 (159–1435)	488 (202–1344)	0.88^*^
TNF (pg/ml) (Median, interquartile range)	304 (146–547)	279 (138–510)	326 (152–592)	0.07^*^
IL10 (pg/ml) (Median, interquartile range)	133 (74–231	145 (82–239)	131 (74–230)	0.75^*^
IgE (IU/ml) (Median, interquartile range)	964 (514–2073)	755 (481–1625)	1105 (540–2248)	0.002^*^

Abbreviations: BMI = body mass index, WHR = waist to hip ratio, FBG = fasting blood glucose, HOMAIR = Homeostasis model assessment for insulin resistance, HsCRP = High sensitive C reactive protein, TNF = tumor necrosis factor, IL10 = interleukin 10, IgE = Immunoglobulin E.

^*^after logarithmic transformation

### Association between STH infection and glucose metabolism (Table 2)

BMI and WHR were lower in infected (22.5 kg/m^2^ and 0.88 respectively) than in uninfected participants (23.2 kg/m^2^ and 0.89), which were independent of age and sex. No differences in FBG glucose concentration were present between subjects with and without STH infection. In comparison with uninfected participants, participants with any STH infection had a trend towards lower HOMAIR but this difference was not statistically significant (mean difference 0.15, p = 0.06).

**Table 2:** Parameters of glucose metabolism parameters in soil-transmitted helminth uninfected and infected participants.

	Mean difference adjusted for age and sex (95% confidence interval)	Mean difference adjusted for age, sex and BMI (95% confidence interval)	Trend analysis # adjusted for age, sex and BMI (95% confidence interval)
BMI (Kg/m2)	-0.6 (-1.2, -0.02), p = 0.04		-0.3 (-0.7, -0.02), p = 0.04
WHR*	-0.01 (-0.02, -0.001), p = 0.02		-0.007 (-0.012, -0.002), p = 0.01
FBG (mmol/L)	-0.05 (-0.3, 0.2), p = 0.7	0.01 (-0.2, 0.3), p = 0.9	-0.08 (-0.22, 0.06), p = 0.3
Insulin (pmol/L)	-4.2 (-14.7, 6.2), p = 0.4	-1.5 (-11.5, 8.5), p = 0.8	-4.9 (-10.3, 0.36), p = 0.07
HOMAIR (index**)	-0.15 (-0.32, 0.01), p = 0.06	-0.10 (-0.25, 0.05), p = 0.2	-0.10 (-0.19, -0.02), p = 0.01

Abbreviations: BMI = body mass index, WHR = waist to hip ratio, FBG = fasting blood glucose, HOMAIR = Homeostasis model assessment for insulin resistance.

* WHR is calculated by waist circumference (cm) / hip circumference (cm)

** HOMAIR index is calculated with HOMAIR formula = fasting serum insulin x fasting glucose / 22.5, using HOMA2 calculator (https://www.dtu.ox.ac.uk/homacalculator/)

# The difference is expressed as increase or decrease in the parameter per increasing number of helminth species per patient (maximum = 3). Insulin and HOMAIR were log-transformed.

**Fig 1:**
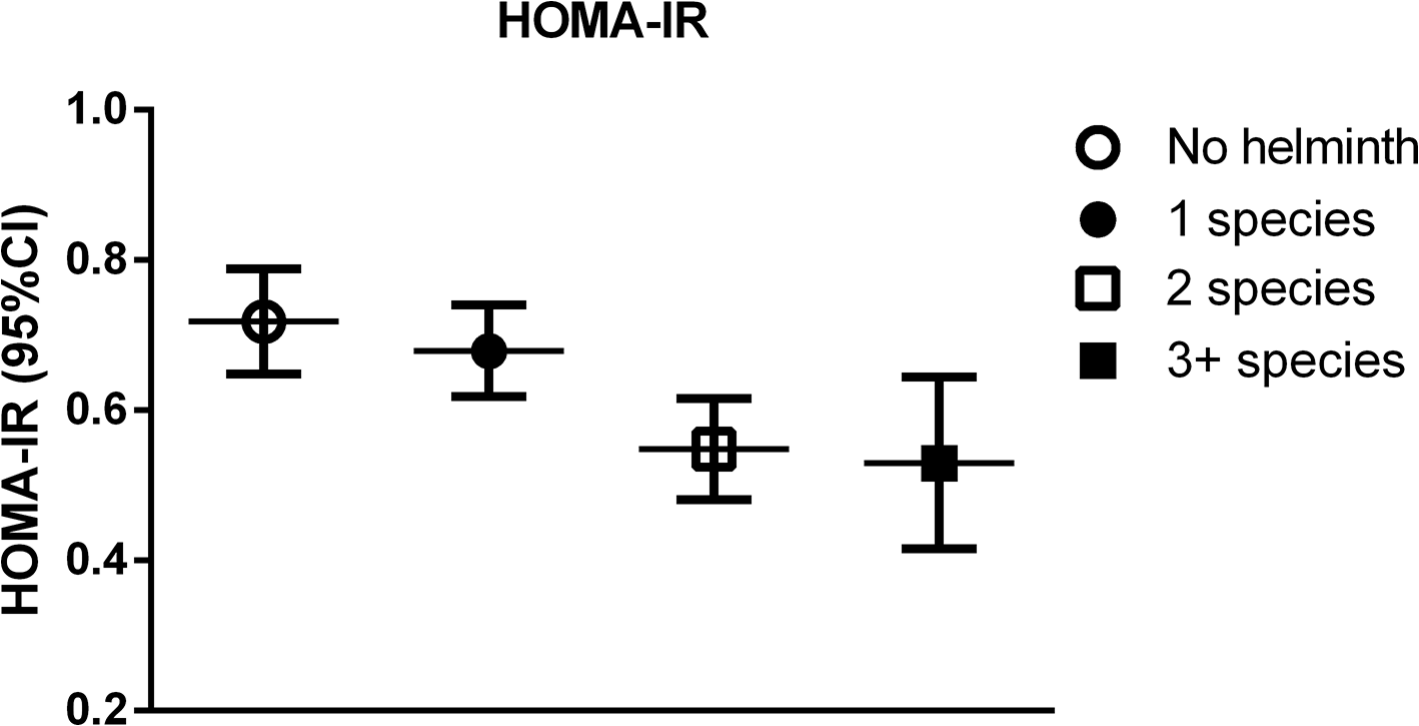
The association of number of soil-transmitted helminth species per subject and Homeostasis Model Assessment for insulin Resistance (HOMAIR). The association of number of helminth species infections with HOMAIR (mean, 95%CI) with correction for age, sex, and BMI. The numbers of participants with none, one, two or at least three species were respectively 161, 189, 98 and 29. The p for linear trend is 0.01. *Adjusted mean for HOMAIR were derived from linear regression. White circle = no soil-transmitted helminth infection; black circle = infected with one soil-transmitted helminth species; white square = infected with two soil-transmitted helminth species; black square = infected with at least three soil-transmitted helminth species.

We found an association between number of STH species per subject and BMI, with a decrease of 0.3 kg/m^2^ for every increase in the number of species (p for linear trend = 0.04, Table 3); a similar association was found for WHR (p for linear trend = 0.01). In an age-, sex- and BMI-adjusted model an association was found between the number of STH species per subject and HOMAIR: for every additional STH species, HOMAIR decreased by 0.10 (p for linear trend = 0.01) (Table 3, Fig 1).

**Table 3:** Immune parameters in soil-transmitted helminth uninfected and infected participants.

	Mean difference adjusted for age and sex (95% confidence interval)	Mean difference adjusted for age, sex and BMI (95% confidence interval)	Trend analysis # adjusted for age, sex and BMI (95% confidence interval)
HsCRP (ng/ml) (n = 496)	-0.00 (-0.13, 0.12), p = 0.96	0.10 (-0.11, 0.13), p = 0.88	-0.02 (-0.08, 0.05), p = 0.60
TNF (pg/ml) (n = 346)	0.10 (-0.00, 0.21), p = 0.06	0.10 (-0.01, 0.21), p = 0.07	0.06 (0.00, 0.12), p = 0.04
IL10 (pg/ml) (n = 346)	-0.01 (-0.10, 0.08), p = 0.82	-0.02 (-0.11, 0.08), p = 0.74	-0.01 (-0.06, 0.04), p = 0.65
IgE (IU/ml) (n = 510)	0.15 (0.06, 0.24), p = 0.002	0.15 (0.05, 0.24), p = 0.002	0.01 (0.05, 0.15), p<0.0001

Abbreviations: HsCRP = High sensitive C reactive protein, TNF = tumor necrosis factor, IL10 = interleukin 10, IgE = Immunoglobulin E. HsCRP, TNF, IL10 and IgE are log-transformed.

# The difference is expressed as increase or decrease in the parameter per increasing number of infections per patient (maximum = 3).

No clear associations were found between STH infections and hsCRP, TNF or IL10 after whole blood stimulation with LPS (TNF-LPS and IL10-LPS). We found a small but positive association between TNF-LPS with increasing number of STH species per subject with the highest TNF-LPS-levels in participants with 3 or more infections (Table 3). This association was not changed after adjustment for age, sex and BMI. No clear association was found between IgE levels and HOMAIR (p = 0.99, crude, p = 0.87, adjusted for age and sex, p = 0.60, adjusted for age, sex and BMI).

We also investigated whether there were differences in the association between the species of STH infections and HOMAIR. We found no individual associations between *N*. *americanus*, *A*. *lumbricoides*, or *T*. *trichiura* (prevalence of *S*. *stercoralis* and *A*. *duodenale* were too low to be considered), with HOMAIR, although these analyses might lack sufficient statistical power.

## Discussion

In this study we investigated the relationship between infection with STH and insulin resistance in a population residing in an area highly endemic for STH. The hypothesis being tested is that STH infections may have a beneficial effect on glucose metabolism, by influencing BMI or their ability to skew immune responses to Th2 and an anti-inflammatory profile. The beneficial influence of STH infections on glucose metabolism has been shown in animal models of T2DM [5]. Mice on high fat diet that were subsequently infected with helminths, became less obese and less insulin resistant which seemed to be in conjunction with maintenance of alternative activated macrophages in adipose tissues [5,16].

In our study, we observed a trend towards lower HOMAIR in subjects with STH infection as compared with uninfected subjects. Furthermore, we found that infection with an increasing number of STH species incrementally enhanced insulin sensitivity. This effect may be partly explained by the lower BMI in STH infected subjects. It should be noted that BMI reference values in Asia are different from Western countries [32]. It is well known that a relationship exists between helminths and energy metabolism. This relationship may be multifactorial and include changes in digestion and decreased absorption of nutrients [21,33]. However, after adjustment for BMI, the negative association between STH infections and HOMAIR persisted, indicating that this association cannot be explained by effects of STH infections on BMI alone. In the analysis using BMI reference values in Asia, we found a significant positive association between the BMI classification and FBG, insulin level and HOMAIR, as expected.

It is tempting to speculate that STH infections in our study may have influenced systemic inflammation which would then lead to improved glucose tolerance. However we found no indication for differences in systemic inflammation between subjects with and without STH infections.

The fact that we did not find statistically significant differences in IL10 levels between infected and non-infected subjects points to the complexity of IL10 regulation, IL10 for instance can be inhibited by insulin, which can have implications for obesity [34].

It should be noted that the magnitude of the effect of STH on HOMAIR in our study is modest. The reduction of 13% in HOMAIR by presence of STH, might reflect the fact that the population is lean and insulin sensitive. Indeed, the low HOMAIR value in our study population as well as the lower BMI and WHR represent the rural character of the area with accompanying healthier lifestyle [35,36].

Alternatively, the difference between currently STH infected and uninfected subjects might be small due to the possibility that the energy balance and immune profiles could still be modulated by previous infections, recent deworming treatment, or sub-microscopic infection. We chose to analyse *A*. *lumbricoides*, Hookworm (*N*. *americanus* and *A*. *duodenale*) and *Strongyloides stercoralis* using qPCR, a method that is more sensitive and reliable than microscopy [31]. However, as we do not have an optimized qPCR method to detect *T*. *trichiura*, while the species is also endemic in the area, we measured *T*. *trichiura* infection using the microscopy method. The fact that we were not able to verify these factors is a limitation of the study.

As mentioned above, STH infection can modulate the host’s immune system but this was not clearly shown in our study participants. An interesting additional explanation may be that that STH affect the gut microbiome which on its turn will also affect the host immune system [37–41].

We acknowledge the limitations of this cross-sectional study, which prevent conclusions on causal relationships between STH infections and T2DM. The population studied was not at risk of T2DM, so potential effects of STH on insulin sensitivity might have been difficult to detect. In addition, no consistent effects of STH on the immune system were found. Furthermore, no data on the history and intensity of helminth infections nor history of deworming are present, which prevents strong conclusions on the relationship between STH infections, the immune system and insulin sensitivity. Furthermore, the fact that only participants were included who provided stool samples may also have led to selection bias.

Using HOMAIR, which is measured in fasting state, may fail to detect early disturbances in insulin sensitivity, which is typically a post-prandial disturbance [42]. An oral glucose tolerance test may therefore have been worthwhile to perform.

It is clear that further and specifically designed investigations are needed to clarify the relationship between innate and adaptive immune responses, inflammation, STH infections and insulin sensitivity in humans.

We acknowledge that the pathogenesis of insulin resistance and diabetes is multifactorial and extremely complex. However, we believe that our study provides interesting data that add a layer of complexity that needs to be taken into account in the relationship between rural-urban transition in LMIC and the development of T2DM.

## Supporting Information

S1 Table Body mass index of study participants in relation to fasting blood glucose, insulin and HOMAIR. (DOCX)
