# Correction: Infection with Soil-Transmitted Helminths Is Associated with Increased Insulin Sensitivity

**DOI:** 10.1371/journal.pone.0136002

**Published:** 2015-08-13

**Authors:** Aprilianto E. Wiria, Firdaus Hamid, Linda J. Wammes, Margaretta A. Prasetyani, Olaf M. Dekkers, Linda May, Maria M. M. Kaisar, Jaco J. Verweij, Bruno Guigas, Felix Partono, Erliyani Sartono, Taniawati Supali, Maria Yazdanbakhsh, Johannes W. A. Smit

The second author is missing an affiliation. Firdaus Hamid is also affiliated with #2, Department of Parasitology, Leiden University Medical Center, 2333ZA, Leiden, The Netherlands.
