# Supplementary material for: Infection with Soil-Transmitted Helminths Is Associated with Increased Insulin Sensitivity
Source: PLoS One. 2015 Jun 10;10(6):e0127746. doi: 10.1371/journal.pone.0127746 (PMC4464734; doi:10.1371/journal.pone.0127746)
Supplement: S1 Table — (DOCX) [file pone.0127746.s001.docx]

**S1 Table. Body mass index of study participants in relation to fasting blood glucose, insulin and HOMAIR.**

| Asian BMI classification (kg/m2) | N = 646  (n, %) | FBG <5.6 mmol/L  N = 262  (n, %) | FBG 5.6-7 mmol/L  N=285  (n, %) | FBG 7-11 mmol/L  N=27  (n, %) | FBG >11 mmol/L  N=10  (n, %) | Insulin  (pmol/L) (mean, SD) | HOMAIR  (mean, SD) |
| --- | --- | --- | --- | --- | --- | --- | --- |
| Underweight (<18.5) | 81 (12.5) | 40 (15.3) | 34 (11.9) | 1 (3.7) | 0 (0) | 22.8 (14.0) | 0.44 (0.28) |
| Normoweight (18.5-22.9) | 277 (42.9) | 135 (51.5) | 111 (38.9) | 4 (14.8) | 2 (20.0) | 35.5 (30.7) | 0.69 (0.58) |
| Overweight (23-24.9) | 127 (19.7) | 46 (17.6) | 56 (19.7) | 7 (25.9) | 4 (40.0) | 47.9 (35.6) | 1.02 (1.27) |
| Preobese (25-29.9) | 135 (20.9) | 35 (13.4) | 73 (25.6) | 11 (40.7) | 3 (30.0) | 57.9 (35.2) | 1.15 (0.73) |
| Obese 1 (30-34.9) | 23 (3.6) | 5 (1.9) | 10 (3.5) | 3 (11.1) | 1 (10.0) | 80.5 (55.1) | 1.57 (1.02) |
| Obese 2 (>35) | 3 (0.5) | 1 (0.3) | 1 (0.3) | 1 (3.7) | 0 (0) | 168.9 (148.4) | 3.22 (2.90) |

Abbreviation: BMI = body mass index, FBG = fasting blood glucose, HOMAIR = homeostatis model assessment index for insulin resistant.

HOMAIR index is calculated with HOMAIR formula = fasting serum insulin x fasting glucose / 22.5, using HOMA2 calculator (https://www.dtu.ox.ac.uk/homacalculator/)

Trend analysis, adjusted for age and sex for FBG (mean, 95% confidence interval) is 0.35 (0.24, 0.46), p<0.0001.

Trend analysis (log-transformed), adjusted for age and sex for insulin (mean, 95% confidence interval) is 0.12 (0.09, 1.14), p<0.0001.

Trend analysis (log-transformed) adjusted for age and sex for HOMAIR (mean, 95% confidence interval) is 0.12 (0.10, 1.44), p<0.0001.
